# Seasonal Patterns of Longevity and Germination in Grass Species Determine the Vegetation Recovery Potential of Degraded Semi‐Arid Savannas

**DOI:** 10.1002/pei3.70229

**Published:** 2026-10-22

**Authors:** Zewdu K. Tessema, Willem F. de Boer, Herbert H. T. Prins

**Affiliations:** ^1^ Department of Natural Resources Management University of Gondar Gondar Ethiopia; ^2^ Wildlife Ecology and Conservation Group Wageningen University Wageningen the Netherlands

**Keywords:** aboveground vegetation, annual grass, communal rangelands, dormancy, heavy grazing, perennial grass, seed longevity, seed viability, soil seed banks, vegetation degradation

## Abstract

Studies indicate that soil seed bank (SSB) could play an important role in triggering the recovery of grass vegetation after degradation. However, the dissimilarity between SSB composition and aboveground vegetation (AGV) is reported in semi‐arid African savannas, which could be due to differences in seed longevity, as determined by seed dormancy and germination. Therefore, the longevity and germination success of 16 grass species collected in semi‐arid savannas of Ethiopia were studied to test whether the poor vegetation recovery of degraded semi‐arid savannas could be attributed to the low germination rate and long dormancy of grass seeds, and whether the difference in species composition between the AGV and SSB could be explained by differences in germination rates between annual and perennial grass species. Our results showed that perennial grass (PG) species had a higher viability and germination rates than the annual grass (AG) species. The mean viability of AG and PG species was 12% (range: 0%–38%) and 46% (20%–90%), respectively. Similarly, the mean germination rates of AG and PG species were 5% (0%–8%) and 33% (6%–90%), respectively. The germination rates of PG species peaked after 8 months since the seeds were placed for germination study. Our study confirmed that differences in germination rates between AG and PG species partly explained the variations in difference in species compositions between the AGV and SSBs. From this study, we concluded that PG species had a high germination rate and a low dormancy indicating that they could not form persistent SSB, and can therefore disappear rapidly after germination, such as under the influence of grazing and trampling, erratic rainfall events, or prolonged dry periods, which is a typical feature of the semi‐arid African savannas.

## 1 Introduction

In tropical semi‐arid savannas, grass species are the dominant herbaceous vegetation with scattered trees and shrubs (Scott et al. 2010; Li et al. 2025). However, intensive grazing, the dominant form of land use in semi‐arid rangelands of eastern Africa (Tessema et al. 2011; Huang et al. 2022), often leads to degradation of the grass cover, causing negative impacts on the ecosystems, livestock production, and livelihoods of the communities (Harris 2010; Li et al. 2025). This degradation is characterized by a reduction in total vegetation cover and palatable plant species, a change in species composition, as well as a reduction in soil quality (Tessema et al. 2011; Snyman 2013; Huang et al. 2022). However, some studies suggest that the return of PG and other species that disappeared under intensive grazing can be facilitated by the SSB and trigger the vegetation recovery (Van Langevelde et al. 2016; Zida et al. 2020), because the SSB serves as a reserve to return to the original vegetation, and regeneration from the SSB has long been considered an important pathway for restoration of degraded vegetation (Huang et al. 2022; Li et al. 2025).

Plant regeneration from seeds is an essential feature of the life cycle of most grass species for the rapid vegetation recovery of degraded ecosystems (El‐Keblawy and Al‐Rawai 2005; Guan et al. 2009). Successful plant recruitment is a function of seed availability, seed germination, and seedling survival (Humphries and Florentine 2022; Tessema et al. 2026). Once the seeds reach the soil surface, the seeds of most species are dormant for some time, after which germination starts, and during the start of germination (Tessema et al. 2026), the seeds are exposed to predation, microbial degradation, or soil burial, depending on species and environmental conditions (Fenner and Thompson 2005; Humphries and Florentine 2022; Benitez‐Malvido 2023). If seeds do not germinate immediately and escape predation and microbial degradation after dispersal, they are usually buried in the soil, which then adds to SSB (Hopfensperger 2007). However, various studies (Snyman 2013; Tessema et al. 2012, 2016; Van Langevelde et al. 2016) showed that the seeds of PG species in the SSB were unable to kick start the vegetation recovery and drive the transition from a degraded vegetation state to vegetation with a PG cover and good fodder value in semi‐arid African savannas. Similarly, semi‐arid rangelands did not recover from heavy livestock grazing even after many years of grazing exclosures (Snyman 2013; Huang et al. 2022; Li et al. 2025). This might be associated with the depletions of grass seeds in the soil (Tessema et al. 2016; Van Langevelde et al. 2016) and/or related to the longevity and germination of seeds in the soil (Humphries and Florentine 2022; Benitez‐Malvido 2023; Tessema et al. 2026).

The SSB composition is determined primarily by seed rain from the AGV directly and hence the above‐and below‐ground species compositions are often similar (Hopfensperger 2007). However, a significant dissimilarity between the composition of SSB and AGV has been noted in terms of palatable PG in semi‐arid African savannas (Tessema et al. 2012, 2016; Van Langevelde et al. 2016), as a result of intensive livestock grazing (Angassa and Oba 2010; Tessema et al. 2011). The dissimilarity is characterized by more PG and woody plants in the AGV under light grazing (Abule et al. 2005; Tessema et al. 2012, 2016) and more AG and forb species in the SSBs under heavy grazing (Solomon et al. 2006; Tessema et al. 2012, 2016). Such dissimilarities can be caused by differences in seed longevity (Tessema et al. 2026), determined by temporal patterns in seed dormancy and germination rates (Baskin and Baskin 2004; Scott et al. 2010; Tessema et al. 2026).

Soil seed bank forming species have seeds with an environmental adaptive strategy that delays germination until specific conditions of the environment are met (Mašková and Poschlod 2022; Moravcová et al. 2022), and how long these seeds can stay buried in the soil while maintaining viability depends on the type of species and the season after initial seed dispersal (Fenner and Thompson 2005; Benitez‐Malvido 2023). The optimum time for seed germination in strongly seasonal climates (i.e., arid and semi‐arid savannas) is as early in the growing season as possible to secure resources such as nutrients and water for growth and reproduction but not so early that survival is unlikely (McIvor and Howden 2000; Moravcová et al. 2022; Benitez‐Malvido 2023). Seed dormancy is one of the main strategies ensuring suitable timing of germination with respect to favorable climatic conditions (Fenner and Thompson 2005; Wesche et al. 2006). The accumulation of seeds in the soil is favored by the dormancy of the seeds, caused by either the presence of embryo dormancy or impermeable seed coat or both (Baskin and Baskin 2004). The seeds of most grass species in tropical savannas are dormant following seed fall in the late wet season but overcome this dormancy during the late dry season when soil temperatures increase (Mott 1978; McIvor and Howden 2000; Tessema et al. 2026). Despite a loss of dormancy during the dry season, germination is usually prevented by a lack of moisture (Scott et al. 2010), after which in response to the first rainfall events of the wet season, seeds germinate in a series of cohorts until no viable seed remain in the soil (Andrew and Mott 1983; Veenendaal et al. 1996).

Understanding the dormancy and germination of grass species is therefore vital to facilitate rehabilitation of degraded areas in arid and semiarid environments (Tessema et al. 2026). Hence, we studied the longevity and seed germination of 16 grass species [4 annuals and 12 perennials] collected at two sites, typical of the semi‐arid savannas of Ethiopia, to understand whether the difference in species composition between the AGV and SSB could be explained by the difference in germination rates between AG and PG species. Based on the above reasoning, we tested the following hypotheses: (i) PG species have a different germination strategy than AG, characterized by a high germination rate and a low dormancy so they do not form persistent seeds in the SSB and thereafter disappear rapidly; (ii) AG species have a low germination and high dormancy rate, so they form persistent seeds in the SSB, and (iii) the dissimilarity in species composition between the AGV and SSB is a reflection of the difference in germination rates between AG and PG species in semi‐arid African savannas.

## 2 Materials and Methods

### 2.1 Location of the Study Areas

The grass seeds for our study were collected in the light and heavy grazed sites both at Abernosa Cattle Breeding Ranch (ACBR: 7^o^47′ N, 38^o^40′ E, 1660–1740 masl) and Awash National Park (ANP: 9^o^20′ N 40^o^20′ E 960–1050 masl), both located in the Ethiopian Rift Valley Ethiopia (Figure 1). Both locations are typical of the semi‐arid savanna grazing systems of Ethiopia, characterized by almost similar rainfall and dry seasons, as well as hot day and night temperatures throughout the year. The mean annual rainfall of ANP was 512 mm and was highly variable among years (coefficient of variation CV = 103%) (Tessema et al. 2016). Its main rainy season is from July–October with a short rainy season from February–April. The mean daily minimum and maximum temperatures were 18°C and 34°C respectively (Tessema et al. 2016). The ACBR has a bimodal rainfall; the short rain is from February–April, followed by a short dry spell, in May and June, and the main rainy season, from July to October, and a long dry period, from November to January. The average annual rainfall of ACBR was 734 mm (CV = 116%). The mean minimum and maximum temperatures were 14°C and 28°C, respectively.

**FIGURE 1:**
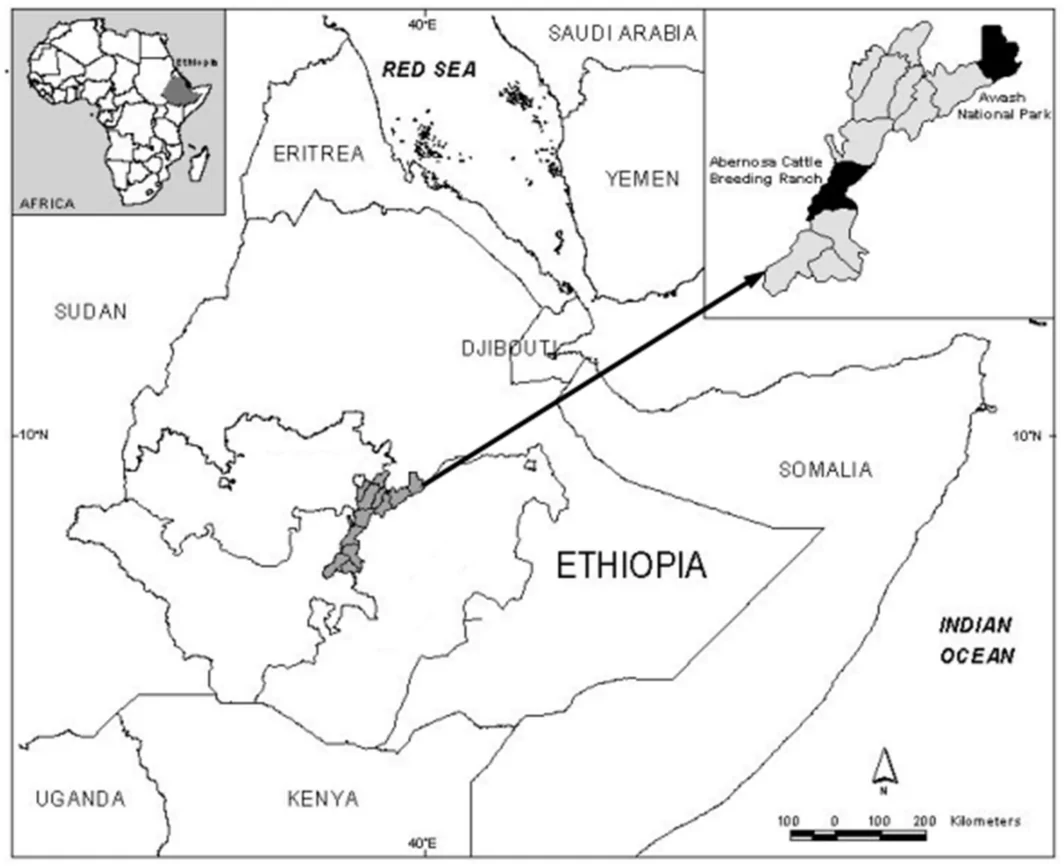
Location of the study areas Abernosa Cattle Breeding Ranch (7^o^47′ N 38^o^40′ E) and Awash National Park (9^o^20′ N 40^o^20′ E) in a semi‐arid savanna grazing systems of Ethiopia.

### 2.2 Procedures of Seed Collection and Germination

Fully matured seeds of 16 grass species [4 annuals and 12 perennials] were collected between the end of October and November 2018. The grass species used for this experiment were found both in the lightly and heavily grazed areas, and species only found in lightly grazed areas based on previous studies (Tessema et al. 2011, 2012, 2016). The AG species included in this study were *Dactyloctenium aegypticum* (DA), *Eragrostis papposa* (EP), *Panicum atrosanguinium* (PA), and *Sorghum verticilliforum* (SV), and the PG species were *Andropogon kelleri* (AK), *Bothriochloa radicans (*BR), *Cenchrus ciliaris* (CC), *Chloris gayana* (CG), *Chrysopogon plumulosus* (CP), *Heteropogon contortus* (HC), *Hyparrhenia hirta* (HH), *Hyparrhenia rufa* (HR), *Panicum coloratum* (PC), *Linthonia nutans* (LN), *Sporobolus pyramidalis* (SP), and *Tetrapogon tenellus* (TT). Prior to the viability and germination studies, the grass seeds were checked for insect attack or other organisms, as well as for their physical damage and intactness. Only intact (undamaged) seeds were used for this study. Seeds were not scarified prior to the viability and germination experiments, except the viability of SV was done with and without scarification since we observed a hard seed coat during our pilot study.

For each grass species, a total of 600 seeds were used for the whole germination study. Hundred seeds of each grass species were sown on a double layer of filter paper, which was kept moist with distilled water in Petri dishes (9 cm diameter), and were placed randomly on a bench in a seed laboratory under room temperature for a period of 28 days (El‐Keblawy and Al‐Rawai 2005). The seed germination test for all grass species was repeated six times, with an interval of 2 months in 1 year, from January–December 2019 at Haramaya University in Ethiopia. The germinated seeds were counted, recorded, and removed every 2 days until the end of the germination time (28 days) (El‐Keblawy and Al‐Rawai 2005). Similarly, 25 seeds of each grass species were used to test the seed viability using a tetrazolium solution (10 g tetrazolium mixed with 1 L of distilled water; AOSA [Association of Official Seed Analysts] 2000) also repeated 6 times with an interval of 2 months for one year similar to the germination test. A seed was considered viable when the embryo part of the seed stained red after immersing the grass seed in a tetrazolium solution overnight.

### 2.3 Data Collection and Analyses

The seed germination rate (GR) and the viability (*V*) for each grass species *i* were calculated as GR_*i*_ (%) = [(∑*n*_*i*_/*N*_*i*_) × 100] and *V*_*i*_ (%) = [((∑*v*_*i*_)/*V*_*i*_) × 100], where ∑*n*_i_ is the cumulative number of seeds germinated per month, *N*_*i*_ is the total number of seeds tested for germination, *V*_*i*_ is the total number of seeds tested for viability, and *v*_*i*_ is the number of seeds with red stained embryo at the end of the tetrazolium test per month (El‐Keblawy and Al‐Rawai 2005). To test for differences in germination rate (%) and viability (%), a two‐way analysis of variance (ANOVA) was carried out using a General Linear Model (GLM) in PASW (version 17), with species and time (months) as fixed effects. Tuckey's Post Hoc test was used to test for differences in viability and germination rates among species and months. The viability and germination percentage data were arcsine transformed to meet the assumption of normality and homogeneous variances prior to carrying out the GLM.

Regression analysis was carried out to explain whether the difference in species composition between the AGV and SSB could be explained by the difference in germination rates between AG and PG species. The abundance data (nm^−2^) of grass species composition used for the regression analysis both in the AGV and SSB was obtained from previous studies as part of the project (Tessema et al. 2012, 2016; Table S2). In addition, to investigate the difference in germination strategy between AG and PG species (Table S1), a Kaplan–Meier survival analysis and a Log—Rank test were used for determining the difference in germination rate over the 28 day periods in each of the 6 months (Kleinbaum and Klein 2005).

## 3 Results

Grass species differed significantly (*F*_15,75_ = 47.072, *p* < 0.001; Figure 2) in seed viability with PG species having a higher viability than AG species. At species level, the seed viability of grass species ranged from 0% (SV without scarification) to 89.7% (HR) (Figure 3). The annual grass SV did not have any viable seeds over the viability test period (1 year). However, the seed viability of SV increased considerably (78%, range: 64%–92%) after physical scarification of its seed coat. The PG species had a higher seed viability than AG (*F*_5,75_ = 10.079, *p* < 0.001; Figure 4a). The PG seed viability generally increased until September and declined thereafter whereas the viability of AG species steadily increased until the end of the 12 month study period.

**FIGURE 2:**
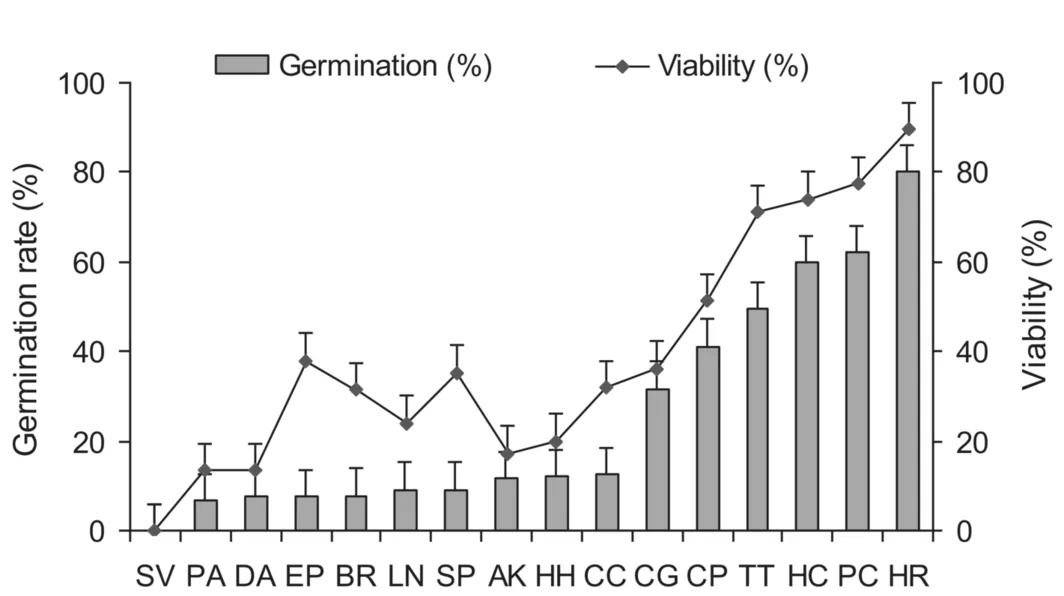
Mean (±95% CI) viability and germination rates (%) of grass species (SV = *Sorghum verticilliforum*, PA = *Panicum atrosanguinium*, DA = *Dactyloctenium aegypticum*, EP = *Eragrostis papposa*), (BR = *Bothriochloa radicans*, LN = *Linthonia nutans*, SP = *Sporobolus pyramidalis*, AK = *Andropogon kelleri*, HH = *Hyparrhenia hirta* , CC = *Cenchrus ciliaris*, CG = *Chloris gayana*, CP = *Chrysopogon plumulosus*, TT = *Tetrapogon tenellus*, HC = *Heteropogon contortus*, PC = *Panicum coloratum*, HR = *Hyparrhenia rufa* ) species collected in a semi‐arid savanna grazing systems of Ethiopia.

**FIGURE 3:**
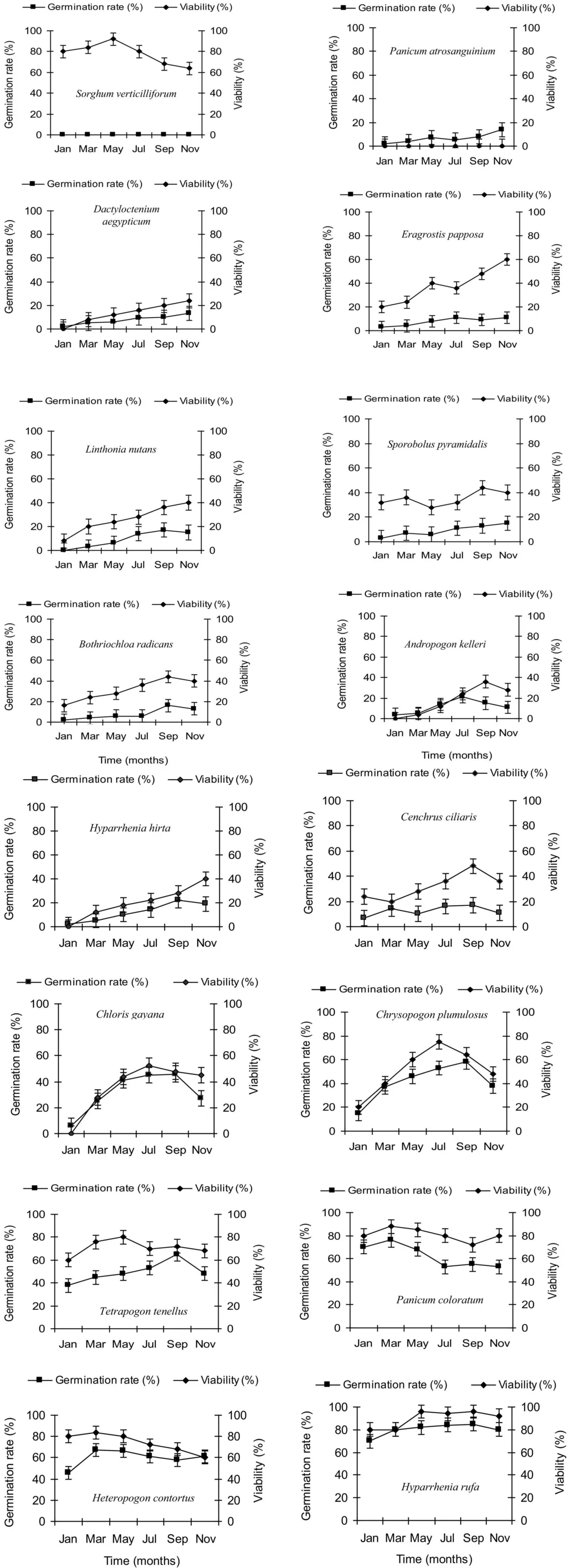
Mean viability and germination rates (±95% CI) over time, from January–December 2019; with an interval of 2 months of annual grass species (*Dactyloctenium aegyticum*, *Eragrostis papposa*, *Panicum atrosanguinium*, and *Sorghum verticilliforum*) and perennial grass species (*Andropogon kelleri, Bothriochloa radicans, Cenchrus ciliaris, Chloris gayana, Chrysopogon plumulosus, Heteropogon contortus, Hyparrhenia hirta, Hyparrhenia rufa, Linthonia nutans, Penicum coloratum*, *Sporobolus pyramidalis* , and *Tetraopogon tenellus*).

**FIGURE 4:**
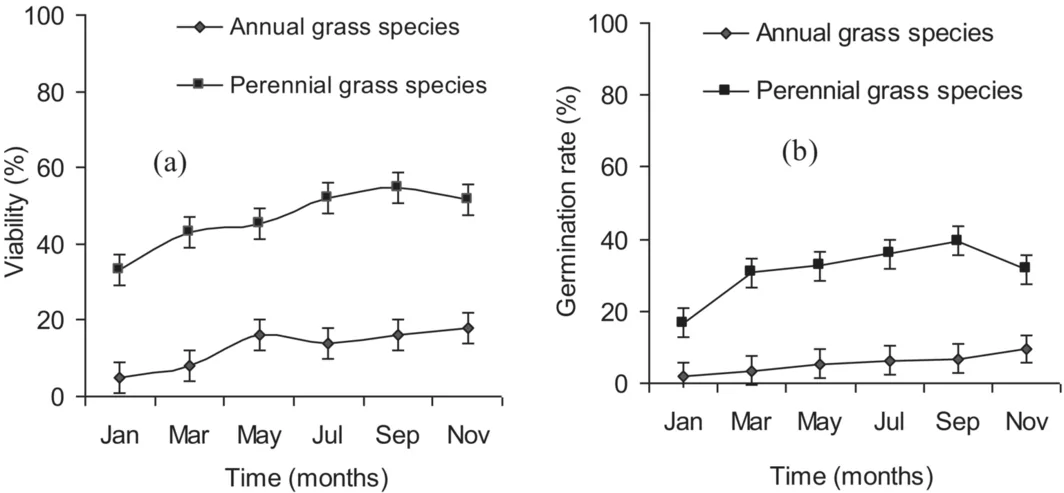
Mean (±95% CI) viability (%; a) and germination rates (%; b) of annual and perennial grass species over time, from January–December 2019 with an interval of 2 months.

The PG species also had a higher germination rate than AG species (*F*_15,75_ = 88.891, *p* < 0.001; Figure 3). The mean germination success (%) of PG species ranged from 7.8% (BR) to 80.2% (HR), whereas for annuals, this value ranged from 0% (SV) to only 7.7% (EP) (Figure 3). Among the PG species, LN, SP, BR, AK, HH, and (CC) still had a higher germination success than all AG species, but their germination rates were lower compared to other PG species, such as CG, CP, TT, PC, HC, or HR (Figure 3).

The germination time (months) had a highly significant effect on the germination rates (*F*_5,75_ = 8.641, *p* < 0.001; Figure 4a). The germination rate of AG species showed an increasing trend until the end of the germination study, whereas the germination rate of PG peaked in September and decreased thereafter (Figure 4b). Looking at the germination success of the individual seeds over the 28 days period, AG species showed a linear trend in germination over time, whereas PG species germinated rapidly in the first days of the experiment and decreased thereafter (Figure 5). Large differences in germination success were observed between AG and PG species, especially in the period from May–June (Log‐Rank test, *X* ^2^ = 116.504; DF = 1, *p* < 0.001) and from September–October (Log‐Rank test *X* ^2^ = 141.000, DF = 1, *p* < 0.001), with a higher germination success for the PG species. Our results confirmed that the difference in germination rates between AG and PG species partly explained the variation in difference between the species composition of the AGV and SSB (Figure 6). For grass species with a mean germination rate of 20%, the germination rates were positively related to the difference in the relative composition of the species between the AGV and SSB (*F*_1,10_ = 8.445, *p* = 0.016; *R* ^2^ = 0.458). According to the results of our regression analysis, the higher the dormancy of the grasses and hence the lower the germination rate, the lower would be the relative contribution of the grass species in the AGV compared to the SSB in semi‐arid savanna of Ethiopia.

**FIGURE 5:**
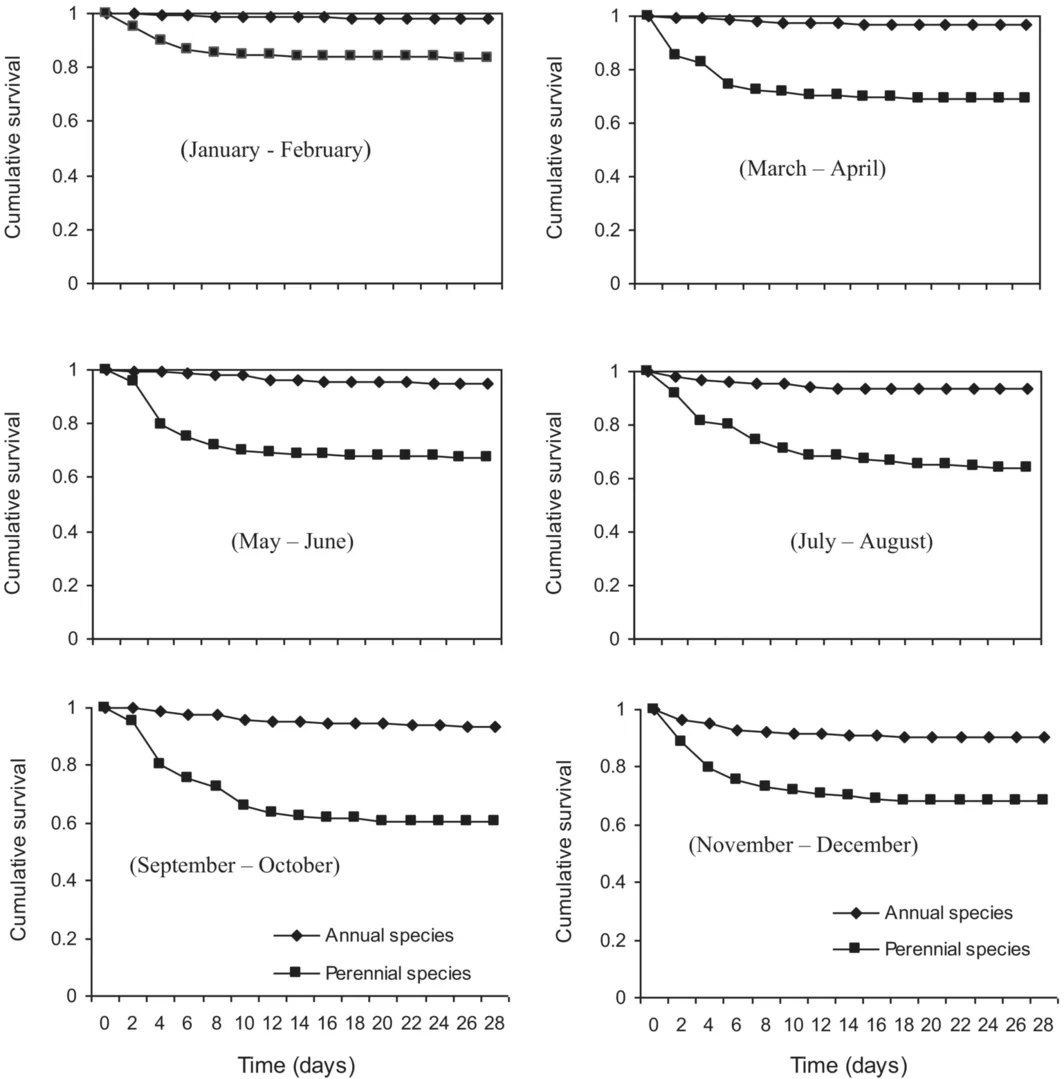
Survival time of seeds for annual and perennial grass species collected in semi‐arid savanna grazing systems of Ethiopia over the 28 days germination trial carried out at six different periods from January–December 2019 with an interval of 2 months.

**FIGURE 6:**
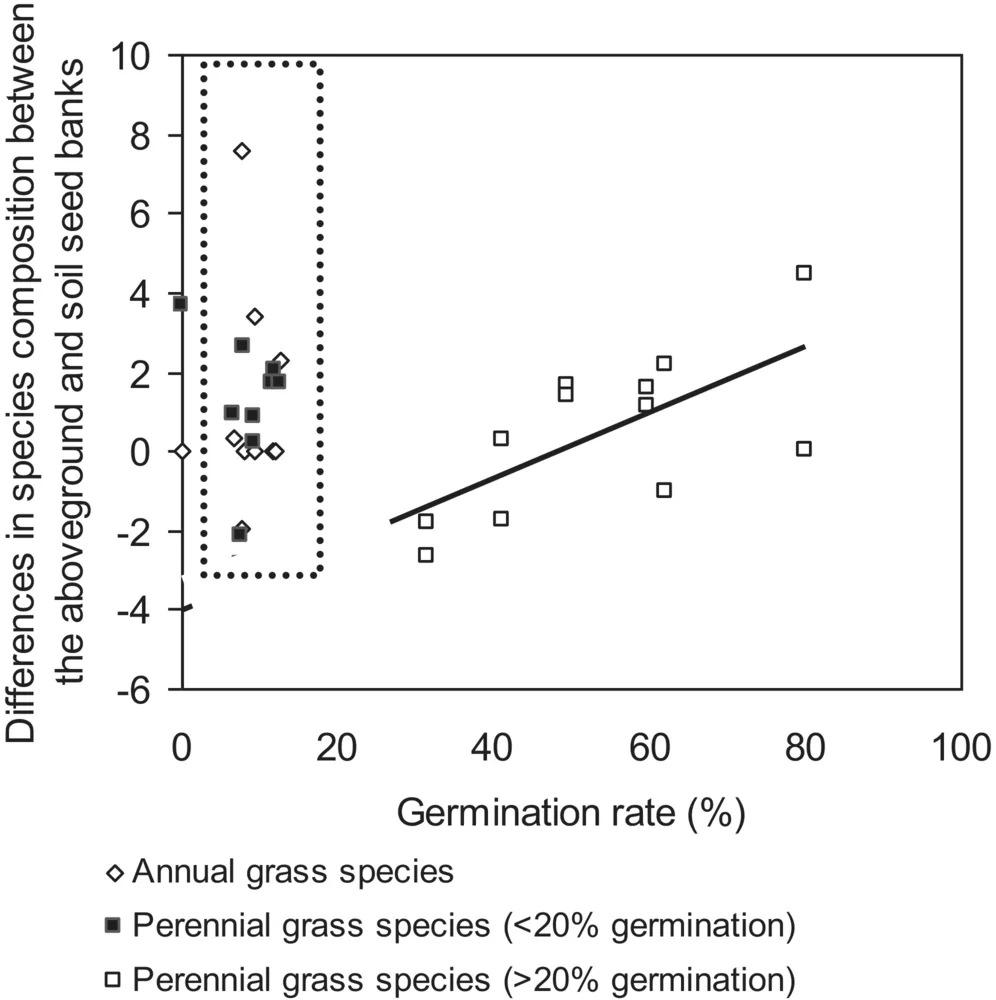
Effect of germination rates on differences in relative composition (%) of grass species between the aboveground vegetation and soil seed banks in semi‐arid savannas of Ethiopia.

## 4 Discussion

There were wide ranges in seed viability and germination rates in grass species over time in our study, indicating grass species with different dormancy and germination characteristics coexist in the study areas. The variability in longevity and germination of the studied grass species could be an opportunity for maintaining the resilience of the ecosystem during variable environmental conditions in semiarid African savannas. In general, AG species had a lower viability and germination rate, which could be explained by a longer seed dormancy (Tessema et al. 2026). Thus, the accumulation of seeds of AG species in the SSB is favored by the dormancy of these seeds, which is probably caused by either embryonic dormancy or the presence of an impermeable seed coat (Benitez‐Malvido 2023). For instance, in our study, the annual species SV drastically increased its viability from 0% to 78% (range: 64%–92%) after physical scarification of the seed coat. According to some studies (Vleeshouwers et al. 1995; Veenendaal et al. 1996; Loddo et al. 2019), dormancy is selected for most annual species as most species are typically found in areas characterized by long dry seasons and unreliable rainfall periods, such as the African semi‐arid savannas.

The germination rates of AG species were low but increased toward the end of the study, indicating that AG species in our study possess a dormancy characteristic (Tessema et al. 2026) in which the development or germination is blocked by one or more factors (Khan and Ungar 1984; Bittencourt et al. 2017). Seed dormancy is a temporary failure of seeds to germinate under particular environmental conditions but initiates germination later by either natural or physical dormancy breaking strategies (Bittencourt et al. 2017; Loddo et al. 2019). For instance, the studied AG species, DA and EP, are good colonizers of bare areas under heavy grazing in the Ethiopian savannas (Abule et al. 2005; Angassa and Oba 2010; Tessema et al. 2011) in degraded areas (Yayneshet et al. 2009) and in other tropical areas of the world (McIvor and Howden 2000; Tefera et al. 2010), which might be due to their long dormancy allowing them to form persistent SSB and germinate when a suitable window arises (Solomon et al. 2006; Tessema et al. 2012, 2026). However, the germination of PG species increased until 8 months, peaked at 10 months, and decreased thereafter at 12 months. The result of our study is in agreement with previous studies, indicating that the seeds of most PG species are dormant following seed dispersal at the end of the growing season but overcome their dormancy during the late dry season due to high soil temperature (Mott 1978; McIvor and Howden 2000).

Most of the PG species (i.e., CP, PC, and HR CP) in our study showed higher germination rates at the beginning of the germination experiment, from January to June, indicating that these species could germinate with the onset of the short rains that usually occur from February–April and the seedlings will probably die due to the short dry spell, which occurs from May–June, in semi‐arid savannas of Ethiopia, where this study was conducted. As a result, PG species, with good fodder values, could be lost most of their seeds in the SSB and the possibility of seeds germinate during the long rainy season, from July–September decreases. According to Benitez‐Malvido (2023), the composition of the SSB depends on the longevity of the seeds of each species under local conditions. However, PG species, such as CP, PC and HR, which had high germination rates were typically found under lightly grazed sites in semi‐arid savanna of Ethiopia (Abule et al. 2005; Tessema et al. 2011, 2012, 2016), indicating that they could be lost under heavily grazed sites after germination due to erratic rainfall, livestock grazing and trampling effects. In a similar fashion, McIvor and Howden (2000) reported that HC, CG and *Chrysopogon* species had a low dormancy during the dry and wet seasons in seasonally dry topical areas of Australia, which is in agreement with our findings.

Several PG species are dormant at seed maturity but they start to break their dormancy during the long dry season, especially when approaching the wet season (Mott 1978). Despite the loss of dormancy in the dry season, germination of PG is usually prevented by a lack of moisture (Scott et al. 2010; Mašková and Poschlod 2022), which is a characteristic of semi‐arid savannas and where seeds germinate in a series of cohorts until no viable seed remains in the soil (Andrew and Mott 1983; Veenendaal et al. 1996). Hence, long‐term dormancy of PG seeds (O'Connor and Everson 1998; Tessema et al. 2026) or their persistent SSB are not common in tropical savannas (Scott et al. 2010; Tessema et al. 2012, 2016).

Persistent seeds are able to prolong their seed dormancy. In our study, AG species had a lower germination rate, and a longer dormancy period before germination. Hence, under the light grazing sites, PG species usually dominate while the heavily grazed sites are dominated by AG and other plants with annual life history (Solomon et al. 2006; Tessema et al. 2011). So, with increasing grazing pressure, palatable PG species of good fodder value are replaced by AG species in the SSB (Tessema et al. 2012, 2016). In general, based on the germination success and time of germination, the grass species in our study could be categorized into 4 groups:

- Group I—The AG, SV with no germination during the entire study period
- Group II—AG species with a relatively high dormancy and low germination rates (< 8%) germinating most often in the long rainy seasons such as PA, DA, and EP;
- Group III—PG species with intermediate germination rates (> 9%–20%) germinating partly in the short rainy season but mostly in the long rainy season such as AK, BR, LN, SP, CC, and HH;
- Group IV—PG species with high germination rates (20%–80%) that break their dormancy in the long dry season, germinating most frequently in the short rainy seasons. As a result, their seedlings are expected to die during the short dry season and ultimately disappear in the SSBs during the long rainy season, and these include CP, CG, TT, PC, HC, and HR.

The African semi‐arid savanna grazing systems are characterized by a large temporal and spatial variation in rainfall (Veenendaal et al. 1996; Tilahun 2006). Moreover, there are two dry spells in semi‐arid savannas of Ethiopia; the long dry season (November–January) and the short dry season (May–June), and two rainy seasons in between (Tilahun 2006). However, most PG species are expected to break their dormancy immediately after seed dispersal during the long dry season and start germination in the short rainy season, which would drastically affect the survival of these perennials. According to Veenendaal et al. (1996), seedling emergence and subsequent survival of PG species may be strongly influenced by the timing and variation in rainfall. A minimum amount of 15–25 mm rainfall has been reported to trigger widespread germination of grass species in arid and semi‐arid grasslands (Andrew and Mott 1983; Veenendaal et al. 1996). However, a single rainfall event, especially if early in the dry season, may be followed by a dry spell increasing the mortality of PG seedlings.

Our study confirmed that differences in germination rates between AG and PG species partly explained the variations in difference in species compositions between the AGV and SSB. The PG species germinated rapidly after initial seed dispersal and declined thereafter, whereas AG species germinated linearly overtime. This shows that AG and PG species have different survival strategies in semi‐arid savannas. According to some studies (Snyman 2013; Tessema et al. 2016), the accumulation of larger quantities of seeds for annual species in the SSB may be favored by the higher dormancy of the seeds (Tessema et al. 2026), which is prolonged by the presence of embryonic dormancy and impermeable seeds coats. Moreover, the residence time of the seeds of annual species in the soil is relatively higher compared to perennials and woody species increasing their viability over a longer period. According to results of our study, PG species may not contribute largely to the formation of persistent SSB in semi‐arid savannas of Ethiopia due to their higher germination rates and lower dormancy characteristics compared to AG species (Tessema et al. 2026), as they tend to have larger persistent SSB. In previous studies (Tessema et al. 2012, 2016; Van Langevelde et al. 2016), AG and forbs usually dominate the SSB, while many perennial species are poorly represented. Generally, AG and herbaceous species contribute most of their seeds in the soil of arid and semi‐arid ecosystems (Li and Zhao 2005), indicating the restoration of PG in arid environments is difficult due to grazing and trampling, shortage and erratic rainfall events, limiting SSB formation, and growth of PG.

## 5 Conclusions

The wide variations in germination rates between AG and PG species revealed that there could be large differences in the establishment of grass seedlings after small rainfall events; PG species germinated rapidly within a few days whereas annuals required more days to germinate. This rapid germination of perennials may particularly increase the likelihood of their establishment in semi‐arid savannas. However, perennials could disappear after germination due to grazing and/or due to long dry periods after germination. Therefore, removal of PG species from the rangeland community in semi‐arid savanna ecosystems for more than 1 or 2 years by heavy grazing is likely to exhaust the seeds available for reestablishment in the SSB, triggering permanent changes to these plant communities unless the species are able to recolonize from elsewhere or are deliberately reintroduced. We believe that this study could provide alarming information to the policymakers and researchers to design appropriate strategies before losing valuable PG species as sources of livestock grazing in the rangelands of Ethiopia in particular and to the rest of the semi‐arid savannas of Africa.

## Funding

The authors have nothing to report.

## Conflicts of Interest

The authors declare no conflicts of interest.

## Supporting information


**Table S1:** Grass seed laboratory germination test over a period of 12 months, within an interval of 2 months, collected in a semi‐arid savannas of Ethiopia.

**Table S2:** Species composition (abundance m^−2^) of annual and perennial grasses both in the soil seed bank and aboveground vegetation, and their difference between aboveground vegetation and soil seed bank under light (LG) and heavy grazing (HG) in semi‐arid savannas of Ethiopia.

## Data Availability

Data used for the article are attached as Tables S1 and S2.
