# Supplementary material for: Seasonal Patterns of Longevity and Germination in Grass Species Determine the Vegetation Recovery Potential of Degraded Semi‐Arid Savannas
Source: Plant Environ Interact. 2026 Oct 22;7(5):e70229. doi: 10.1002/pei3.70229 (PMC13689716; doi:10.1002/pei3.70229)
Supplement: Supplementary file 1 [file PEI3-7-e70229-s002.docx]

**Table S2.** Species composition (abundance m^-2^) of annual and perennial grasses both in the soil seed bank and aboveground vegetation, and their difference between aboveground vegetation and soil seed bank under light (LG) and heavy grazing (HG) in semi-arid savannas of Ethiopia.

|  | Soil seed bank | | Aboveground vegetation | | Differences in species composition between aboveground vegetation and soil seed bank | | Germination rate (%) |
| --- | --- | --- | --- | --- | --- | --- | --- |
| Species | HG | LG | HG | LG | HG | LG |  |
| *Andropogon kelleri* | 0 | 0 | 0 | 1.72 | 0 | 1.72 | 11.7 |
| *Bothriochloa radicans* | 0 | 1.5 | 0 | 4.12 | 0 | 2.63 | 7.8 |
| *Cenchrus ciliaris* | 0 | 0.8 | 2.31 | 1.69 | 2.31 | 0.92 | 12.5 |
| *Chloris gayana* | 2.6 | 3.1 | 0 | 1.34 | -2.64 | -1.76 | 31.7 |
| *Chrysopogon plumulosus* | 3.7 | 6.8 | 1.91 | 7.11 | -1.74 | 0.36 | 41.2 |
| *Dactyloctenium aegypticum* | 3.3 | 5.7 | 10.90 | 2.89 | 7.60 | -2.81 | 7.5 |
| *Eragrostis papposa* | 5.5 | 3.6 | 3.59 | 1.53 | -1.92 | -2.11 | 7.7 |
| *Heteropogon contortus* | 0 | 0 | 1.60 | 1.14 | 1.60 | 1.12 | 59.8 |
| *Hyparrhenia hirta* | 0 | 0 | 0 | 2.02 | 0 | 2.02 | 12 |
| *Hyparrhenia rufa* | 0 | 0 | 0 | 4.50 | 0 | 4.47 | 80.2 |
| *Linthonia nutans* | 0 | 0.5 | 0 | 1.40 | 0 | 0.87 | 9.2 |
| *Panicum atrosanguinium* | 1.1 | 1.2 | 1.41 | 2.1 | 0.35 | 0.96 | 6.7 |
| *Panicum coloratum* | 1.5 | 0.8 | 0.45 | 2.96 | -1.01 | 2.18 | 52 |
| *Sorghum verticilliforum* | 0 | 0 | 0 | 3.7 | 0 | 3.70 | 0 |
| *Sporobolus pyramidalis* | 0 | 2.5 | 3.4 | 2.7 | 3.4 | 0.25 | 9.2 |
| *Tetrapogon tenellus* | 0 | 0 | 1.7 | 1.4 | 1.6 | 1.4 | 49.5 |
